# White matter pathlength maps from diffusion-weighted MRI tractography for radiotherapy target planning in glioblastoma

**DOI:** 10.1007/s11060-026-05789-9

**Published:** 2026-09-25

**Authors:** Michael Wahl, Christopher H. Chapman, Olivier Morin, Kesshi Jordan, Roland G. Henry, Susan M. Chang, Javier E. Villanueva-Meyer, Pratik Mukherjee, Philip Theodosopoulos, Michael W. McDermott, Mitchel S. Berger, Penny Sneed, Steve E. Braunstein, Janine M. Lupo

**Affiliations:** 1 Department of Radiation Oncology, University of California, Box 0226, 505 Parnassus Ave, 008, San Francisco, CA 94117 USA; 2 Department of Neurology, University of California, San Francisco, CA USA; 3 UCSF/UC Berkeley Graduate Program in Bioengineering, San Francisco & Berkeley, CA USA; 4 Department of Radiology and Biomedical Imaging, University of California, San Francisco, CA USA; 5 Department of Neurological Surgery, University of California, San Francisco, CA USA; 6 Present Address: St. Charles Cancer Institute, Bend, Oregon USA; 7 Present Address: Kaiser Permanente, Dublin, CA USA; 8 Present Address: Baptist Health Miami Neuroscience Institute, Miami, Florida USA

**Keywords:** Glioblastoma, Diffusion-weighted MRI, Tractography, Radiation therapy planning

## Abstract

**Purpose:**

Conventional radiotherapy target delineation for glioblastoma (GBM) includes an isotropic expansion from gross tumor visible on anatomic MRI to an empirically defined clinical target volume (CTV) for coverage of microscopic infiltrative disease. GBM spreads preferentially along white matter tracts, but this information has not previously been systematically incorporated into radiotherapy planning. We investigated using white matter tractography from diffusion-weighted MRI (dwMRI) to inform target delineation.

**Methods:**

Thirteen patients with GBM underwent 55-directional dwMRI at the time of post-operative radiation planning MRI. Whole-brain tractography was performed, and streamlines passing within 5 mm of gross disease were used to generate maps representing white matter path length from gross disease. Clinical target volumes were generated using tractography (CTV_tract_) and conventional isotropic expansion (CTV_isotropic_). MRI at time of GBM recurrence was registered to the radiation planning MRI, and coverage of the recurrence volume was compared between CTV_tract_ and CTV_isotropic_.

**Results:**

CTV_tract_ demonstrated non-isotropic expansion of the primary tumor along regional white matter tracts while respecting natural anatomic boundaries. CTV_tract_ volumes using a 2 cm path-length were a median of 67 cc smaller (-19%) than the paired 2 cm CTV_isotropic_ volumes (*p* = 0.003). 10/13 recurrence volumes were included in CTV_isotropic_, while 12/13 recurrence volumes were included in CTV_tract_.

**Conclusion:**

Tractography can be used to define CTV that may provide superior coverage of areas at risk of recurrence while simultaneously reducing target volume.

## Introduction

Glioblastoma (GBM) is the most common primary malignant brain tumor in adults [1], with a poor prognosis despite decades of effort to improve treatment. While radiation after initial surgery is well-established in the management of GBM [2], optimal target delineation for radiotherapy remains controversial, with multiple proposed techniques but a paucity of data for any particular approach [3]. In general, accepted techniques rely on delineating visible disease on standard anatomic MRI sequences, then applying a 1.5–3 cm isotropic expansion to generate a clinical target volume (CTV) for radiotherapy planning [4–6]. Historically, most patients have recurred within the high-dose region of radiation (in-field) [7], irrespective of the technique employed to define the treatment volume, mitigating further efforts to modify target delineation. However, more modern radiotherapy approaches employing concurrent temozolomide have demonstrated changes in recurrence patterns, with recent studies showing recurrence rates of up to 40% outside the high-dose volume in patients with methylguanine-methyltranferase (MGMT) promoter methylation, which confers increased sensitivity to temozolomide [8–11]. While in-field recurrences remain the dominant pattern of failure, there is a subset of patients for whom regional microscopic disease potentially remains undertreated.

While advanced imaging techniques such as Magnetic Resonance Spectroscopic Imaging (MRSI) [12–14] and molecular PET tracers like ^18^F-FET-PET and ^11^C-MET-PET [15–19] have demonstrated potential to more accurately localize infiltrative disease for RT targeting, this literature is confined to retrospective analyses or simulations, lacks prospective validation in clinical trials, or frequently ignores progression beyond the boundary of the lesion that is hyperintense on T2-weighted MRI. Even more recent phase II single-arm prospective studies that have incorporated advanced imaging to guide treatment, only use advanced imaging to guide where to boost, not define the extent of the CTV [12, 19–24]. Algorithms that can detect microscopic disease by forecasting the pattern of future tumor progression, facilitating early, localized detection of subclinical recurrence, could significantly enhance treatment strategies, especially RT. Although a few studies have explored machine and deep learning-based approaches incorporating multiparametric MR images to predict progression [25–28], these approaches either did not incorporate knowledge of surrounding normal brain white matter anatomy and/or often neglected to consider progression beyond the original T2-hyperintense lesion.

Histopathologic and pre-clinical studies indicate that GBM does not spread isotropically, but rather preferentially along large white matter pathways [29]. Proximity of the tumor to white matter tracts and evidence of surrounding white matter destruction has also been associated with a poor prognosis [30]. However, the propensity for white matter invasion as a route of spread has not yet been incorporated systematically into target delineation for GBM. Diffusion-weighted MRI (dwMRI) is a technique that measures the variable diffusivity of water molecules, which can be anisotropic in the presence of organized white matter microstructure. Tractography uses this information to model the trajectory of white matter fiber bundles in the brain, and prior work in the form of small pilot or feasibility studies has suggested that dwMRI tractography [31, 32], metrics quantified by advanced dwMRI acquisitions [20–22, 33–37], and atlases of tractography-derived white matter tracts [38] may help identify areas of eventual disease recurrence. We have previously developed a tool for white matter pathlength map generation seeded from a lesion boundary using dwMRI tractography [39]. We hypothesize that this algorithm has potential to systematically identify routes of GBM spread to inform radiotherapy treatment volumes. The goal of this exploratory study is to determine the feasibility of using this clinically applicable dwMRI tractography-based algorithm for anisotropic radiotherapy target delineation and evaluate the ability of the technique to more accurately identify areas at risk of initial tumor recurrence than conventional isotropic radiotherapy volumes.

## Methods

### Patients and treatment

Demographic and clinical information for the 13 patients included in this retrospective analysis is shown in Table 1. To be eligible, all patients underwent maximum possible tumor resection followed by post-operative radiation planning MRI including dwMRI tractography within 5 weeks of initiating RT. The median age at diagnosis was 61 years (range 46–68). Twelve patients were isocitrate dehydrogenase (IDH) wild-type. Five patients were MGMT promoter methylated, five were unmethylated, and three did not have methylation status available. All patients received concurrent and adjuvant temozolomide with radiotherapy to a dose of 60 Gy in standard fractionation as standard of care,^2^ and were followed with MRI scans every 2 months at our institution until development of a non-disseminated recurrence that was subsequently confirmed as true recurrence either by surgery or repeated scans. The protocol was approved by our institutional review board for human subjects research.

**Table 1:** Demographic and clinical information for the 13 patients included in the patterns of failure analysis

Patient	Age	Gender	EOR	IDH	MGMT	TMZ	Time to recurrence (months)
1	64	M	GTR	wt	U	Y	5
2	57	M	STR	wt	U	Y	9
3	60	M	STR	wt	M	Y	6
4	64	M	STR	mut	M	Y	9
5	49	M	STR	wt	NA	Y	20
6	66	F	STR	wt	M	Y	20
7	68	M	STR	wt	M	Y	11
8	48	F	STR	wt	U	Y	11
9	61	M	STR	wt	NA	Y	6
10	54	M	GTR	wt	U	Y	16
11	44	F	STR	wt	NA	Y	18
12	61	M	GTR	wt	M	Y	20
13	46	M	STR	wt	U	Y	11

EOR = extent of resection; GTR = gross total resection; STR = subtotal resection. IDH mutation designated by wt (wild-type) or mut (mutated), MGMT methylation status designated by U (unmethylated), M (methylated) or NA (not available)

### Image acquisition

Radiation planning MRI acquired on a 3.0T GE MR750 scanner utilizing an 8-channel phased-array head coil included standard anatomical T1-weighted pre- and post-contrast images (3D BRAVO sequence with an isotropic voxel size of 1.0 mm, TR/TE of 7.2/3.2ms, TI=450ms, a flip angle of 12°, and a 256 × 256 matrix prescribed over a 240–256 mm sagittal FOV covering the entire cranium), and T2-FLAIR MR images (3D CUBE FLAIR sequence with 1.0 to 1.2 mm isotropic voxel resolution, a TR = 6,000–7,600ms, a TE = 90-130ms, TI = 1,850-2,100ms, and 240–256 mm sagittal FOV). High angular resolution diffusion imaging (HARDI) with the 55 directions, b=2000s/mm^2^, 1 b_0_ image, 1.09 × 1.09mm^2^ in-plane resolution, and 2 mm slice thickness was also acquired in 7–8 min acquisition time. Planning CT images were acquired at a 1.25 mm slice thickness, a 512 × 512 matrix, and 250 mm FOV.

### Data processing and tractography

The HARDI datasets were first corrected for motion and eddy current distortion using the FMRIB Software Library (FSL) and the gradient table rotated accordingly [40, 41]. A tensor model was fit to the corrected data using the Diffusion Imaging in Python (DIPY) [42] open-source package to calculate fractional anisotropy (FA) for the purposes of gross anatomical reference, but the fiber tracking was performed with a “higher order” Q-ball model to ensure adequate representation of crossing regions. White matter on the pre-operative MRI, defined as all voxels with a FA greater than 0.15, was tracked at a density of one seed per voxel. Q-ball residual bootstrap fiber tracking was performed using an implementation of DIPY to generate streamlines connecting a set of points in millimeter space [43]. During residual-bootstrap tractography, principal fiber orientation was estimated at each step by computing a bootstrapped orientation distribution function (ODF) and identifying the peaks. The constant solid angle variant of the ODF function was used [44, 45]. ODFs were fit using even order spherical harmonic functions up to order 4. ODF peaks less than 45° from a larger peak and small peaks with values less than one-fourth of the ODF maximum were excluded. The principal fiber orientations from the bootstrap ODFs provided the distribution of fiber tracking directions. Tracking was terminated by minimum FA of 0.15 and maximum angle of 60° [46].

### Target delineation

Target delineation followed the 2016 ESTRO-ACROP guidelines [4]. The gross tumor volume (GTV) was delineated as the post-surgical tumor bed and enhancing regions on T1 post-contrast images plus any additional abnormal mass-like, non-enhancing T2 FLAIR hyperintense signal. All patients were treated with conventionally defined target volumes, with the clinical target volume (CTV) generated by a 2 cm isotropic expansion of the GTV. To account for patient motion and inter- and intra-fraction uncertainty in the tumor location due to variations in patient setup, a standard planning target volume (PTV) was generated by either a 3 or 5 mm isotropic expansion of the CTV. The PTV was prescribed 60 Gy with an objective of 95% target coverage with the prescription dose.

### Creation of white matter path length maps

All HARDI tractography streamlines passing within 5 mm of the GTV were selected to generate a subset of streamlines representing the white matter anatomy in the region of the tumor. These streamlines were then converted into a scalar map with the value of each voxel representing the minimum white matter path length between the voxel and the GTV [39]. The path length function implemented in DIPY computes the shortest path along any streamline between a given ROI and each voxel [42].

### Creation of CTV volumes

The white matter path length maps were imported to the radiation treatment planning system (RayStationV5.0.2, RaySearch Laboratories, Stockholm, Sweden) in the same frame of reference as the dwMRI. The planning CT scan was rigidly registered to the MR images. Three CTV_tract_ were created for each patient by thresholding the white matter path length maps for values of 1, 2, and 3 cm path lengths from the GTV, plus Boolean union with the GTV itself. A 5 mm expansion was finally applied to the results of the union to compensate for registration errors, anatomical changes, and limit the formation of small contour islands. For comparison, standard CTV_isotropic_ was created for each patient by 2 cm isotropic expansion from GTV limited at the skull as per guidelines [3]. Volume differences between CTV_tract_ and CTV_isotropic_ were compared within patients using pairwise Wilcoxon rank-sum test.

### Patterns of failure analysis

For all 13 patients, the follow-up MRI demonstrating radiographical recurrence was rigidly registered to the dwMRI imaging space. The region of enhancing gross recurrence volume (GTV_recurrence_) was defined at contrast-enhancing lesion at the time of progression, delineated using the pre- and post-contrast T1-weighted images. The GTV_recurrence_ was then characterized according to its location in relationship to the CTV_isotropic_ and CTV_tract_ volumes. A “central” recurrence was defined as GTV_recurrence_ located entirely within the original GTV, an “in-field” recurrence as the GTV_recurrence_ located entirely within the CTV, a “marginal” recurrence as the GTV_recurrence_ overlapping but extending outside the CTV, and a “distant” recurrence as the GTV_recurrence_ located entirely outside the CTV. Differences in recurrence coverage classification between CTV_isotropic_ and CTV_tract_ were tested for statistical significant using Fisher’s exact test.

## Results

### Tractography target delineation

**Fig. 1:**
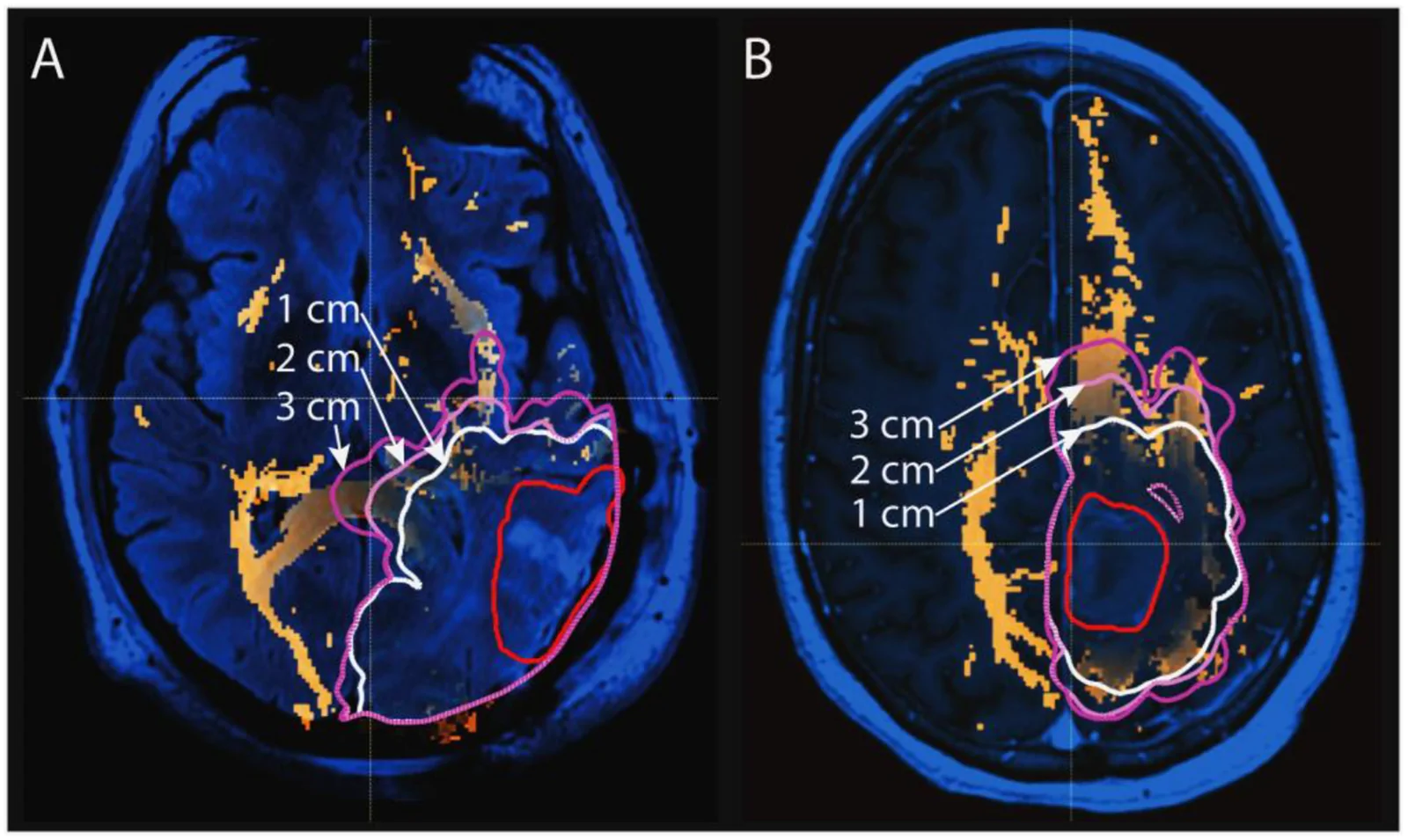
Anisotropic CTV expansions for two patients (**A**: patient 3, an occipital-temporal lesion, **B**: patient 7, a mostly parietal lesion, from Table 1) using white matter path length maps displayed in orange over post-contrast T1 weighted MRI in blue

All 13 patients underwent target delineation with the dwMRI-based target definition using path length expansions of 1, 2 and 3 cm. Figure 1 displays two examples of the white matter path length maps obtained by our algorithm and how the expansion length affects the final CTV_tract_. In all 13 cases, the CTV_tract_ followed white matter pathways consistent with local white matter anatomy. For example, in Fig. 1A, the tractography expansion followed the natural anatomic boundaries but crossed to the contralateral hemisphere via the splenium. In Fig. 1B, the expansion followed the superior cingulum bundle.

### CTV volumes

Table 2 shows volumes per patient for CTV_isotropic_ (2 cm) and CTV_tract_ with 1, 2, and 3 cm expansions. The pairwise CTV volumes were significantly smaller for CTV_tract_ using 1 cm and 2 cm path length expansions. For 1 cm, the mean volume decrease was 136 cc (39%; *p* = 0.0002). For 2 cm, the mean volume decrease was 67 cc (19%; *p* = 0.003). For CTV_tract_ using a 3 cm path length expansion, there was no significant change in target volume (5 cc decrease, 1%; *p* = 0.4).

**Table 2:** Recurrence coverage and volumes per patient for CTV_isotropic_ (2 cm) and CTV_tract_ with 1, 2, and 3 cm expansions

Patient	CTV_isotropic_ central/in-field	CTV_tract_ central/in-field			CTV_isotropic_ volume (cc)	Volume difference CTV_isotropic_ - CTV_tract_		
		1 cm	2 cm	3 cm		1 cm	2 cm	3 cm
1	Y	Y	Y	Y	228	107 (47%)	68 (30%)	18 (8%)
2	Y	Y	Y	Y	395	166 (42%)	99 (25%)	24 (6%)
3	Y	Y	Y	Y	347	153 (44%)	108 (31%)	62 (18%)
4	Y	N	Y	Y	605	254 (42%)	139 (23%)	24 (4%)
5	Y	N	Y	Y	654	177 (27%)	111 (17%)	59 (9%)
6	Y	Y	Y	Y	510	163 (32%)	0 (0%)	-133 (-26%)
7	N	N	N	N	186	78 (42%)	28 (15%)	-26 (-14%)
8	N	N	Y	Y	292	53 (18%)	-29 (-10%)	-29 (-10%)
9	Y	N	Y	Y	298	122 (41%)	54 (18%)	-12 (-4%)
10	Y	Y	Y	Y	203	83 (41%)	35 (17%)	-18 (-9%)
11	Y	N	Y	Y	263	145 (55%)	89 (34%)	26 (10%)
12	N	N	Y	Y	292	108 (37%)	67 (23%)	26 (9%)
13	Y	Y	Y	Y	438	162 (37%)	105 (24%)	48 (11%)
Summary	10/13	6/13	12/13	12/13	362	136 (39%)	67 (19%)	5 (1%)
P-value						*p* = 0.0002	*p* = 0.003	*p* = 0.4

Summary line includes proportion of recurrences covered, mean volume (cc), and mean volume difference (cc) with average percentage difference in parentheses

### Patterns of failure

**Fig. 2:**
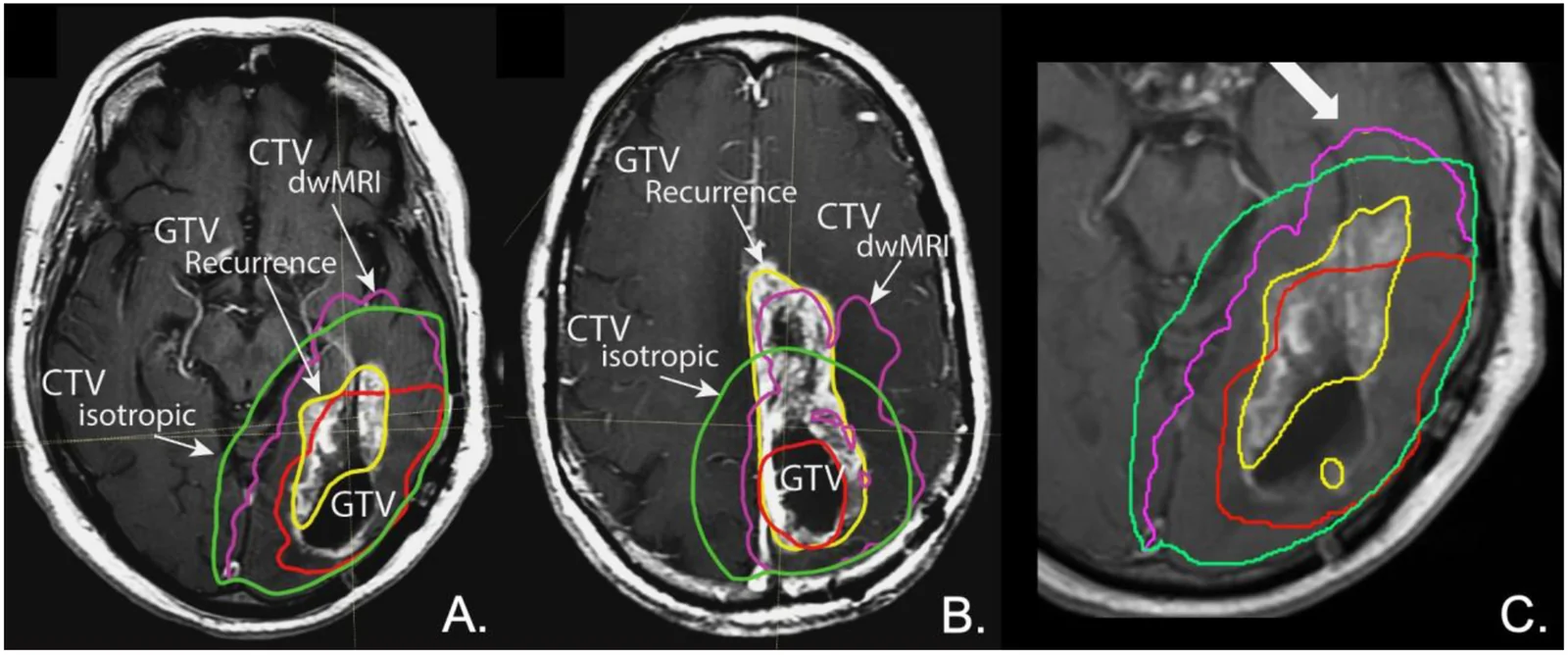
Comparison of the isotropic CTV_isotropic_ (in green) and 2-cm margin anisotropic CTV_tract_ (in magenta). The GTV to CTV expansions in relation to the radiographic recurrence are displayed for 3 different patients of the studied cohort. (**A**) Example of an infield recurrence (patient 3). (**B**) Example of a marginal recurrence (patient 7). (**C**) T1 post-contrast at time of recurrence for patient 12 from Table 1, demonstrating recurrence volume (yellow) covered by both 2 cm CTV_tract_ (magenta) and CTV_isotropic_ (green), but with 2 cm CTV_tract_ overall sparing more brain. The original GTV volume is in red. Arrow indicates recurrent tumor extension along inferior longitudinal fasciculus where the CTV_tract_ captures the overall direction of the progression

The median time to recurrence was 12 months (range 5–20 months). Table 2 lists whether the recurrence for each patient was encompassed (central or in-field) within the CTV_iso_ or the CTV_tract_ for 1, 2 and 3 cm expansions. Of the 5 patients known to have MGMT unmethylated tumors, 4/5 recurrences were covered by CTV_isotropic_, whereas 3/5 recurrences with MGMT methylated tumors were covered by CTV_isotropic_. For all patients, the 1 cm CTV_tract_ expansion did not cover 4recurrences covered by CTV_isotropic_. However, for 2 and 3 cm CTV_tract_ expansions, all recurrences within CTV_isotropic_ were covered by CTV_tract_. Figure 2A shows an example subject (patient 3) who experienced a recurrence encompassed entirely within CTV_isotropic_ and CTV_tract_ with 2 cm expansion, although CTV_tract_ volume was 108 cc (31%) smaller. Figure 2B shows an example subject (patient 7) with a recurrence not completely covered by any CTV definition, although a larger proportion of the recurrence was covered by CTV_tract_ with 2 cm expansion despite a 28 cc (15%) smaller volume. Figure 2C shows an example subject (patient 12) with a recurrence extending along the inferior longitudinal fasciculus that was completely covered by both 2 cm CTV_tract_ and 2 cm CTV_isotropic,_ though the CTV_tract_ definition spared more unaffected brain. Table 3 lists the proportions of central, in-field, marginal, and distant recurrences using the 2 cm isotropic expansion and a 2 cm white matter path length expansion. Despite the trending numeric differences in the proportions, these differences did not reach statistical significance in our limited sample size (*p* = 0.6).

**Table 3:** Proportion of central, in-field, marginal, and distant recurrences for isotropic 2 cm expansion and 2 cm dwMRI-based expansion

Recurrence Type	CTV_isotropic_	CTV_tract_
Central	3 (23%)	3 (23%)
In-field	7 (54%)	9 (69%)
Marginal	3 (23%)	1 (8%)
Distant	0 (0%)	0 (0%)

## Discussion

The optimal technique for target delineation for radiotherapy for glioblastoma remains controversial. Current clinical approaches generally rely on an isotropic expansion of 2 cm from gross disease, derived primarily from a foundational study showing that 90% of recurrences as seen on CT were within a 2 cm isotropic margin of the initial tumor [47]. While historical studies demonstrate that the vast majority of recurrences occur within this 2 cm margin, more recent studies of patients with a relatively favorable prognosis receiving systemic therapy show up to a 40% rate of marginal or distant recurrence, suggesting that current treatment volumes may inadequately cover regions at risk for harboring microscopic disease [8–10]. In addition, the use of a large isotropic expansion potentially encompasses a large volume of normal brain tissue, raising the risk of radio-necrosis and long-term neurocognitive decline as a result of radiotherapy [48].

In this study, we developed a technique for target delineation based on regional white matter tract anatomy, known to be a route of spread for GBM, in an effort to both improve coverage of areas at risk for tumor spread and to minimize the overall treatment volume. Target volumes generated using this technique were significantly smaller than volumes based on a 2 cm isotropic expansion, yet provided better coverage of areas of eventual disease recurrence. We specifically observed two cases in which the tumor recurrence was clearly infiltrating along a large white matter tract in a region that was included in our tractography target volumes but not in a standard radiotherapy target volume. We thus demonstrate both the feasibility of this approach and its potential to improve coverage of regions at risk for microscopic disease without increasing toxicity. Based on this limited dataset, white matter path length of 2 cm appears to improve coverage of recurrences while reducing overall target volumes, though further study is needed to optimize the parameters of the white matter path length expansion.

Prior studies have demonstrated that diffusion tractography identifies routes of tumor spread and explored techniques for using dwMRI to define radiation target volumes [20–22, 29–38, 49, 50]. Many of these studies included small numbers of patients, did not compare the resultant target volumes to those used clinically, or did not perform a systematic pattern-of-failure analysis to determine the potential clinical utility of the technique. Furthermore, several of these techniques typically relied on approaches that could not be as easily implemented clinically [20, 21, 34, 37, 49, 50]. Our study provides the basis for a systematic approach for incorporating tractography into GBM radiotherapy planning.

There are notable limitations to this small exploratory case study. Patient numbers were insufficient to demonstrate statistically that our technique decreased the proportion of marginal or distant recurrences compared to standard techniques. Our patterns-of-failure analysis, while suggestive of improved coverage of regions of recurrence, was observational in nature and should be considered as hypothesis-generating. The small sample size also limited the ability to confidently optimize both the parameters for performing tractography and the thresholds to maximize recurrence coverage while minimizing target volume, prohibiting any quantification of accuracy or predictive modeling. Although our findings will crucially need to be validated and refined in a larger prospective study, they support the proof-of-concept and future collection of such imaging data prior to RT and demonstrate the feasibility and potential for incorporating white matter pathlength maps from dw-tractography into predictive models of tumor recurrence for future anisotropic CTV delineation. Secondly, our finding that the dwMRI tractography-based targets provided qualitatively better coverage of tumor recurrence volumes does not necessarily imply that treatment using these volumes would have prevented these recurrences. Since ~ 63–87% of patients experience recurrence within the high-dose radiotherapy region depending on the study, it remains to be seen whether modifying radiotherapy volumes using this technique would yield a clinical impact on recurrence patterns without a prospective clinical trial. Although this technique may be of utility as an approach for refining the uniform 2 cm-margin beyond the T2-lesion or delineating “high-risk” target volumes for future dose-escalation studies, a formal analysis of how our approach translates into actual sparing of organs at risk and normal brain tissue through dosimetric statistics and DVH parameters in a larger cohort is a necessary next step.

In conclusion, we have developed a technique for incorporating information about regional white matter anatomy based on tractography into target delineation for GBM and demonstrated feasibility of implementation on a small exploratory cohort of 13 patients. Our findings suggest that such a technique may decrease treatment volumes compared to conventional techniques, while potentially improving coverage of areas at risk for tumor recurrence. This raises the possibility that this may be a strategy worthy of further exploration in the future for improving local control with radiotherapy without increasing toxicity. Our results motivate further study in a larger prospective cohort to validate our observations, optimize the technique, and determine the clinical efficacy of this approach both in terms of sparing organs at risk and prolonging survival.

## Data Availability

Data Availability: The imaging dataset used in this study cannot be shared publicly due to patient privacy and ethical restrictions. However, the results generated from this study can be made available from the corresponding author upon reasonable request.
